# Increase in Interfacial Adhesion and Electrochemical Charge Storage Capacity of Polypyrrole on Au Electrodes Using Polyethyleneimine

**DOI:** 10.1038/s41598-019-38615-6

**Published:** 2019-02-18

**Authors:** Kyung-Geun Kim, Sung Yeol Kim

**Affiliations:** School of Mechanical Engineering, Kyungpook National University, Daegu, 702-701 Republic of Korea

## Abstract

High-performance devices based on conducting polymers (CPs) require the fabrication of a thick CP-coated electrode with high stability. Herein, we propose a method for enhancing the interfacial adhesion strength between a gold electrode and an electropolymerized polypyrrole (pPy) layer by introducing a polyethyleneimine (PEI) layer. Although this insulating layer hinders the initial growth of the pPy layer on the Au surface, it improves the adhesion by up to 250%. Therefore, a thick layer of pPy can be fabricated without delamination during drying. X-ray photoelectron spectroscopy shows that the PEI layer interacts with the Au surface via polar/ionic groups and van der Waals interactions. Scanning electron microscopy reveals that the cohesion of the pPy layer is stronger than the interfacial adhesion between the PEI layer and the pPy layer. Importantly, the electroactivities of pPy and its dopant are unaffected by the PEI layer, and the electrochemical storage capacity of the pPy layers on the PEI-coated Au electrodes increases with thickness, reaching ~530 mC/cm^2^. Negative potential sweep is detrimental to pPy layer adhesion: pPy layers on a bare Au electrode peel off instantly as the potential is swept from 0.2 to −0.7 V, and most of the charge stored in the layer becomes inaccessible. In contrast, pPy layers deposited on PEI coated Au electrode are mechanically stable and majority of the charge can be accessed, demonstrating that this method is also effective for enhancing electrochemical stability. Our simple approach can find utility in various applications involving CP-coated electrodes, where thickness-dependent performance must be improved without loss of stability.

## Introduction

Conducting polymers (CPs) such as polypyrrole (pPy) are attracting considerable interest because their redox activity, conductivity, and biocompatibility are useful for various applications, including electrochemical energy storage devices, actuators, and neural interfaces. To fabricate high-performance CP electrodes, it is often desirable to deposit a thick CP layer on the electrode because its performance is highly dependent on this layer’s thickness; for instance, the areal energy/power densities (Wh/cm^2^, W/cm^2^) or actuating force of a CP electrode are proportional to its thickness^1,2^.

However, it is challenging to develop a thick CP layer because of the low interfacial adhesion strength between the CP layer and the underlying electrode. Accordingly, the CP layer can easily peel off from the electrode during fabrication and operation. One strategy to overcome this delamination issue is to roughen the electrode surface by either etching or electroplating its surface^3^. Although mechanical interlocking between the overlaying CP layer and the rough metal surface has been shown to improve interfacial adhesion^3,4^, the CP layers were only a few microns thick. Additionally, the etching process could expose an electrochemically reactive underlying layer (such as Ti), making the CP-coated electrode unstable during long-term application. An alternative strategy involves the formation of covalent bonds between the electrode surface and the pPy layer using modified pyrrole monomers^5,6^. However, this method requires additional effort to synthesize the monomers, and some covalent bonds such as Au surface and thiolated pyrrole cannot be used for electrochemical cycling of the CP layer due to their instability^6^. A recent study showed that the use of a bioinspired adhesive molecule, dopamine, as a dopant for pPy resulted in good interfacial adhesion^7^. However, this method cannot be extended to other functional dopants.

To address the delamination issue, we have developed a new strategy that utilizes an adhesion-enhancing layer between the CP layer and the metal electrode. Herein, we examined a high-molecular-weight branched polyethyleneimine (PEI) as a candidate material for such an adhesion-enhancing layer because of its good adhesion to metals^8^, charged polyelectrolytes^9^, and cells^10^ via van der Waals or ionic/polar group interactions. Moreover, its chain flexibility and large free volume are suitable for accommodating an incipient electropolymerized CP layer.

In this study, we used pPy doped with a redox-active molecule, namely, indigo carmine (IC), as a model CP layer, because it showed good electrochemical performance for energy storage applications and the thickness of the pPy layer was limited to the submicron scale^11^. Our approach improved significantly the adhesion between the pPy layer and the electrode surface. Importantly, the physical characteristics (e.g., morphology, thickness, and charge dependence) and the electroactivity of the pPy layer were not affected by the presence of the PEI layer. Therefore, this simple strategy allowed us to develop thicker pPy layers without compromising their performances. We validated the effectiveness of our method by demonstrating pPy layers with enhanced areal capacities and electrochemical performances.

## Materials and Methods

### Preparation of electrode surface

Single crystal Si (100) wafers with a 100-nm-thick layer of SiO_2_ were used as substrates for the experiments. An e-beam evaporator (SRN 200, Sorona) was used to prepare the Au working electrode by evaporating the metal layers of Ti (10 nm) and Au (50 nm) on one side of the Si wafer. All the Au electrode surfaces were cleaned sequentially under sonication with acetone, ethanol, and deionized (DI) water for 5 min. O_2_ plasma treatment (Plasma Cleaner, PDG-32G, Harrick Plasma) was performed for 2 min on the Au electrode. For PEI (MW = 750,000 g mol^−1^, Sigma Aldrich) adsorption, the electrode was immersed in a 0.5 mg/mL PEI solution for 24 h. For the preparation of thick polymer-coated Au electrodes, a plasma-treated Au electrode was coated with a layer-by-layer assembly of a positively charged polymer (linear polyethyleneimine, LPEI) and a negatively charged polymer (poly(acrylic acid), PAA (MW = 250,000 g mol^−1^)^9^. The layer by layer assembly process is shown in Fig. S1. A plasma-treated Au electrode was first immersed in solution of LPEI for 15 min, followed by immersion of the electrode three times in DI water for 3, 1, and 1 min to remove excess LPEI that is loosely bound to the surface. Then, the electrode was immersed in a solution of poly(acrylic acid) (PAA) and subsequent washing as mentioned above. This deposition process was repeated 5 times to obtain a thick polymer coating on the Au surface.

### Characterization of electrode surface

The amount of PEI adsorption on the gold surface was measured using a quartz crystal microbalance (QCM) (QCA-922, Seiko EG&G). The PEI layer was adsorbed onto one side of Au quartz (QA-A9M Au), which was assembled into dip cells (QA-CL3). The frequency was measured and compared under a constant relative humidity of 30 % and temperature of 23 °C. The QCM results for PEI-O_2_ were tested four times, while those of PEI Au were tested three times, after which the mean and standard deviations were calculated. X-ray photoelectron spectroscopy (XPS) measurements were carried out on the Au surfaces within an area of 100 μm × 100 μm (Quantera SXM, ULVAC-PHI). The XPS spectra of C 1 s, N 1 s, and Au 4 f regions were fitted using a Gaussian function after background subtraction. The surface morphologies of the species absorbed on the Au surface were characterized using atomic force microscopy (AFM, NX20, Park Systems).

### Electrodeposition and characterization of pPy layer

A thin film of pPy was electrodeposited onto the surface-modified Au-Si wafer (1.5 cm × 0.5 cm area) by applying a constant potential of 0.70 V (vs. Ag/AgCl) in an aqueous solution containing 200 mM pyrrole and 25 mM indigo carmine (IC, Sigma Aldrich)^11^. The thickness of the pPy layer was determined by controlling the charge per unit area. All thickness profiles and roughness characteristics of the pPy samples were obtained using a Dektak 150 instrument. Cyclic voltammetry (CV) was performed in 0.2 M HCl solutions at various potential windows and scan rates. After electrodeposition of the pPy layer on the Au electrode, the electrode was rinsed with DI water and dried under a fixed relative humidity (30 %) and temperature (23 °C). The surface morphologies of the delaminated pPy layer and the Au surfaces after delamination were observed by field emission scanning electron microscopy (FE-SEM) (S-4800, Hitachi High-Technology).

## Results and Discussion

**Figure 1:**
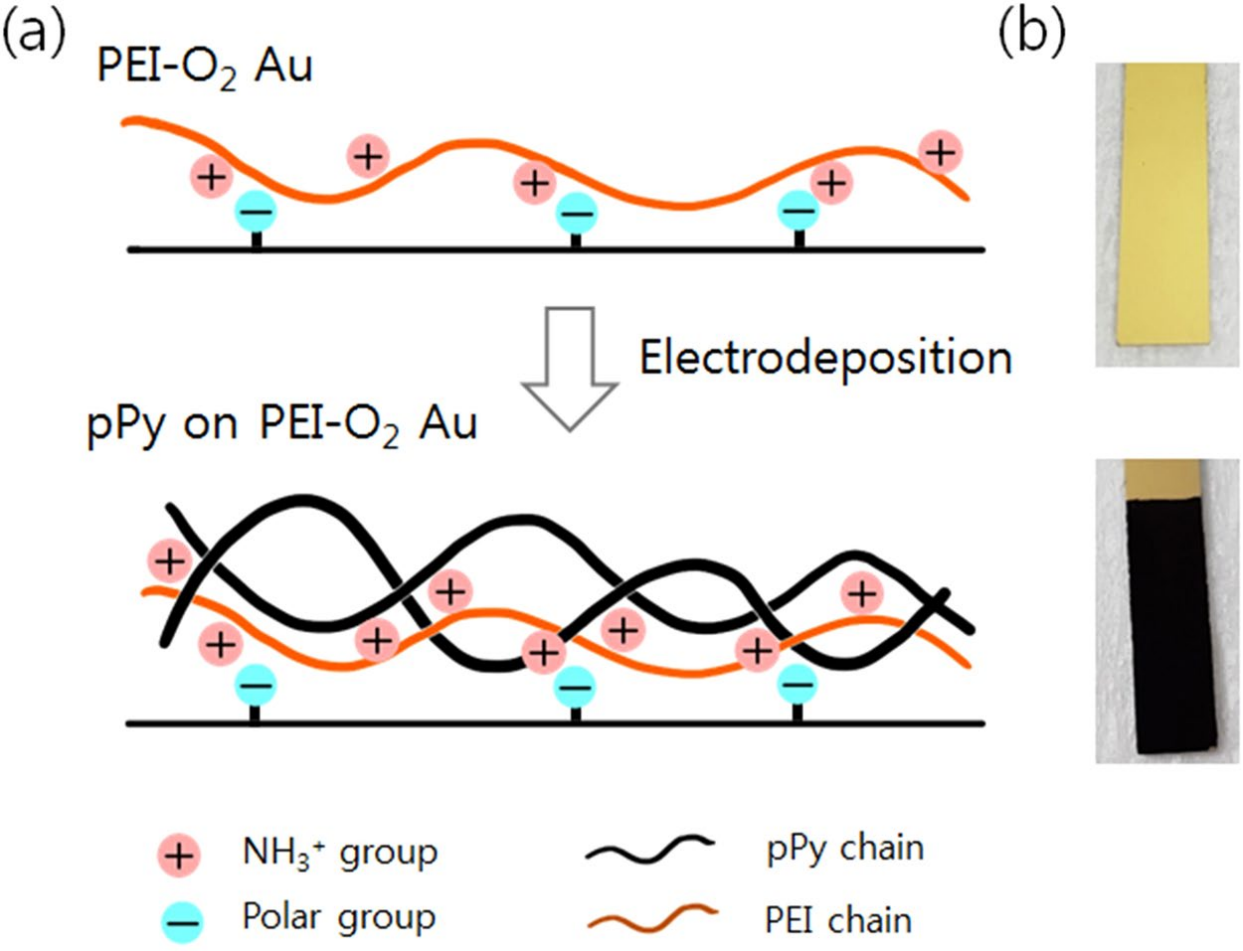
Schematic diagrams of the (**a**) PEI-O_2_ Au electrode and pPy-coated PEI-O_2_ Au electrode after electrodeposition of the pPy layer. The Au surface was first treated with O_2_. plasma before coating the PEI layer. (**b**) Digital images of the Au electrode surface before and after electrodeposition of the pPy layer.

Figure 1 shows our strategy for enhancing the adhesion between the electrodeposited pPy layer and the Au electrode. First, the electrode surface is treated with O_2_ plasma to produce polar functional groups (e.g., carbonyl) on the surface^12^. Second, a PEI layer (orange line) physisorbs onto the plasma-treated electrode via electrostatic interactions involving the positively charged groups of PEI and the polar groups on the electrode surface. After application of the PEI layer, pPy (black line) is then electrodeposited onto this surface (PEI-O_2_ Au). Figure 1(b) shows the digital images of the PEI-O_2_ Au surface before and after the pPy electrodeposition. The PEI-O_2_ Au surface was indistinguishable from the bare Au surface to the naked eye; by contrast, after the electrodeposition, the surface was covered with a black coating of pPy.

**Figure 2:**
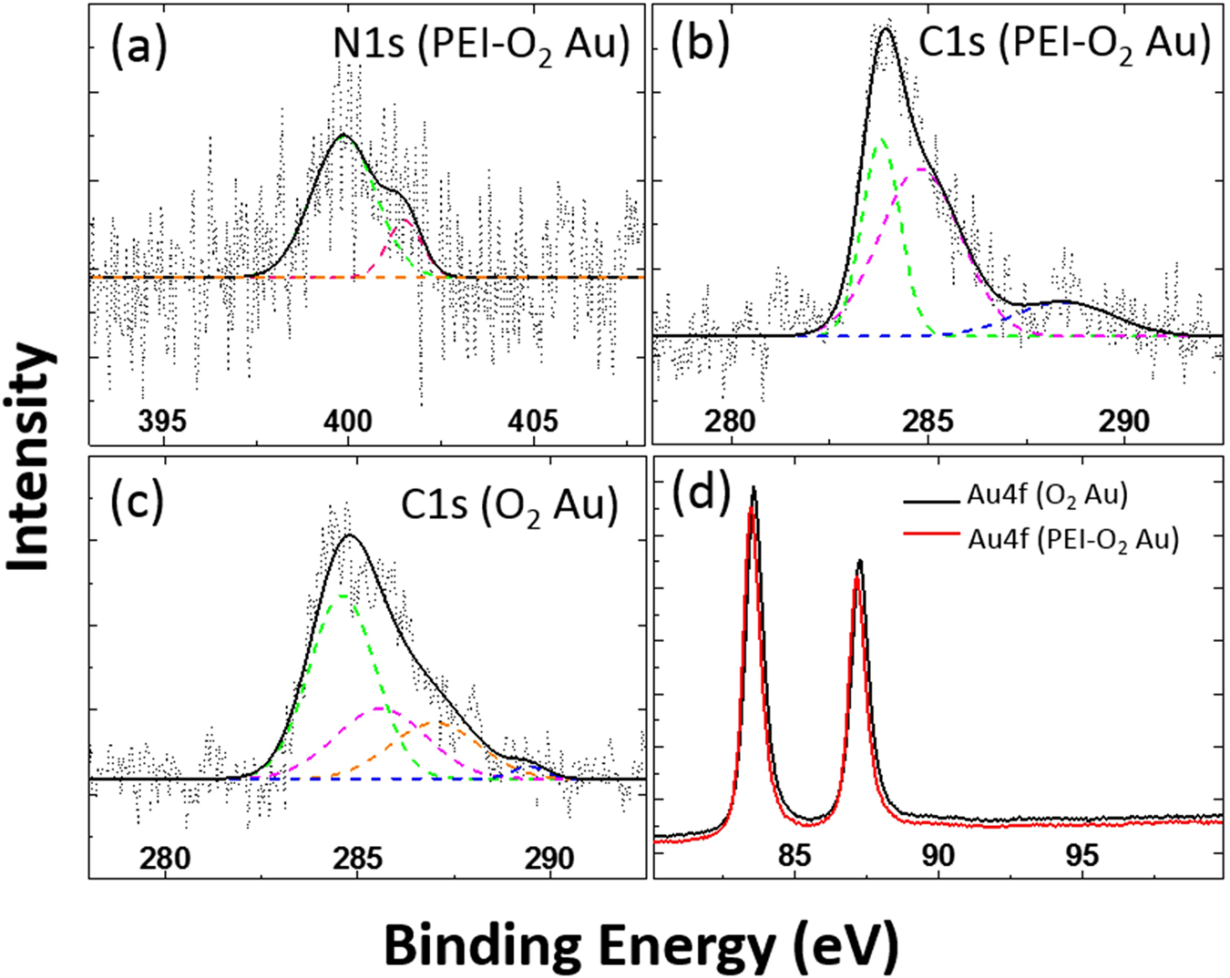
XPS spectra of (**a**) N 1 s, (**b**) C 1 s of a PEI-O_2_ Au electrode, and (**c**) C 1 s of an O_2_ Au electrode. XPS spectra of (**d**) Au 4 f peaks of O_2_ Au and PEI-O_2_ Au electrodes.

In order to investigate the interactions between the PEI and the O_2_ plasma-treated Au surface, XPS was performed on the Au surface before and after the coating with the PEI layer. After coating, new peaks assigned to PEI appeared: N 1 s peaks at 401.5 and 399.9 eV, corresponding to N^+^ and NH/NH_2_ species, respectively (Fig. 2(a)), and a C–N peak at 286.9 eV (Fig. 2(b)). Additionally, the C = O and Au 4 f peaks shifted by 1.0 and −0.15 eV with respect to their original position in the spectrum of the uncoated Au surface, respectively (Fig. 2(c,d)). The slight negative shift of the Au 4 f peaks indicated that the gold surface was reduced by the non-protonated amino groups of PEI^13^. The positive shift of the C = O peak after PEI adsorption indicates the interactions between C = O and the positively charged amino groups of PEI^14,15^. These results show that the PEI layer interacts with both the carbonyl groups present on the treated surface and the Au metal surface.

**Figure 3:**
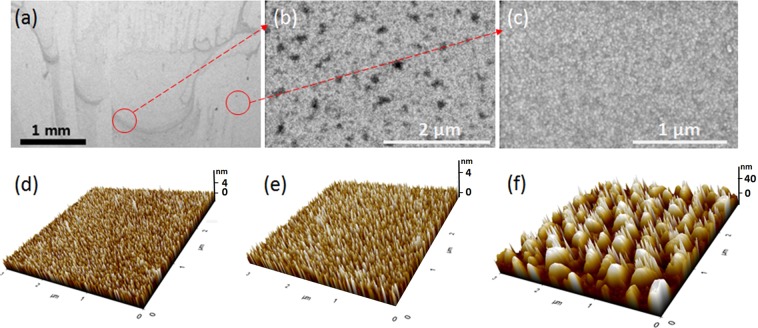
(**a**) Scanning microscopy images of the PEI-O_2_ Au electrode at (**a**) low magnification and (**b**,**c**) high magnification; (**b**,**c**) show dark and bright regions in (**a**), respectively. Atomic force microscopy images of (**d**) bare Au, (**e**) PEI-O_2_ Au, and (**f**) thick polymer coated Au.

We quantified the amount of PEI layer adsorbed onto the O_2_ plasma-treated Au electrode using QCM. The amount was 0.64 ± 0.252 μg/cm^2^, indicating that a PEI layer of 6.4 nm thickness adhered to the Au surface, assuming its density to be 1.03 g/cm^3^ ^16^. This thickness is much greater than that of a PEI monolayer coating (~0.6 nm)^14^, which suggests aggregation of the PEI layer. Scanning electron microscopy (SEM) confirmed that the O_2_ plasma-treated Au electrode was covered with a non-uniform coating of PEI. As shown in Fig. 3(a), dark irregular patches of PEI covered a portion of the electrode surface after the adsorption of PEI; this region has multiple PEI dots on the surface, although most of the area appears similar to the uncoated Au surface (Fig. 3(b,c)). Therefore, a small fraction of the surface appeared to be coated with PEI when visualized by SEM, and the remaining surface was relatively unaffected. After plasma treatment on Au surface, gold oxide is formed, carbon contamination is removed, and polar groups (e.g. CO) appear^14,15^. It has been reported that gold oxide and the sites of polar groups are non-uniform and discontinuous on the surface^17^. The affinity of PEI to different surface sites would be different, and therefore, PEI adsorption should be non-homogenous. AFM revealed that the roughness of this remaining surface was slightly lower than that of the uncoated Au surface (Table S1 and Figure 3(d–f)). This result suggests that this region was also affected by the PEI since the application of a PEI coating on a substrate generally produces smooth surfaces^14^.

**Figure 4:**
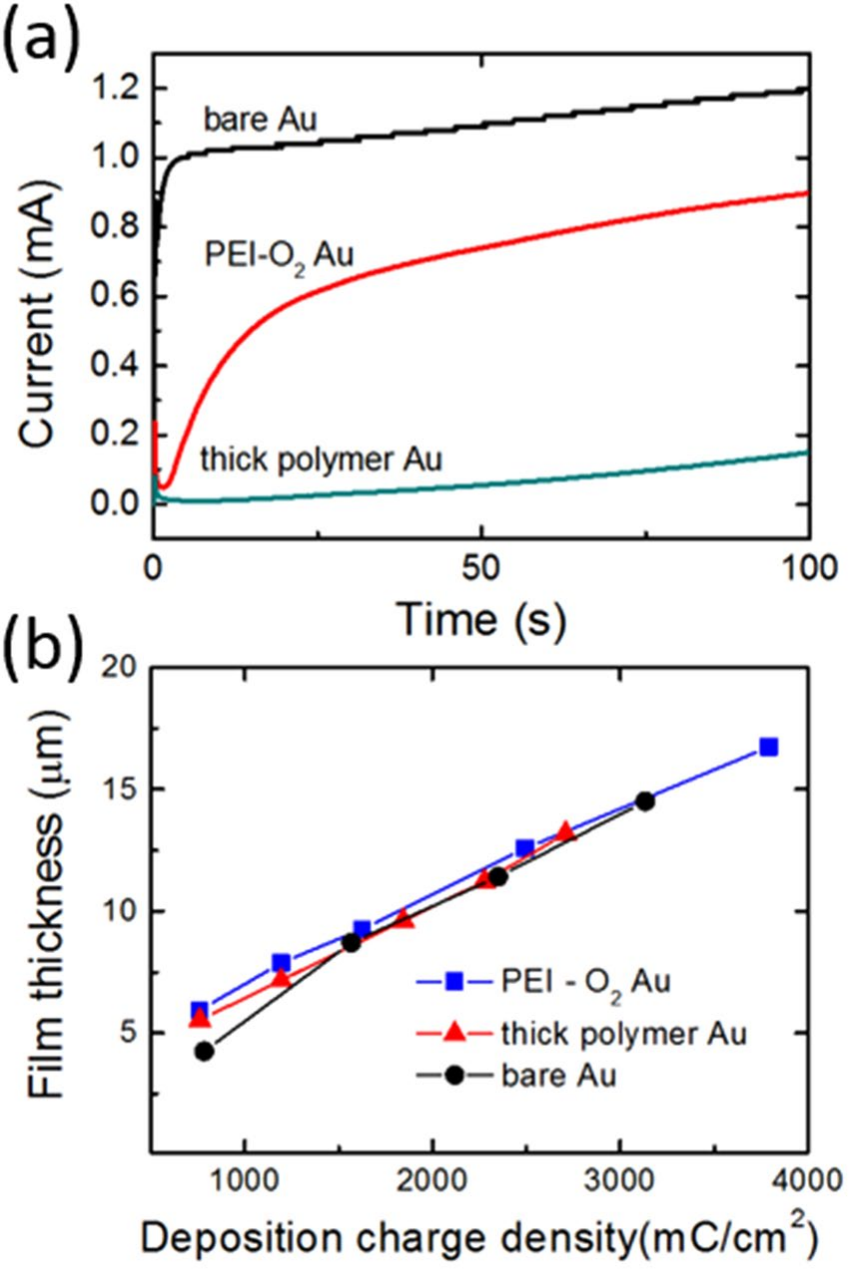
Chronoamperograms of the pPy layers during their electrodeposition on Au electrodes. (**b**) Relationship between the pPy layer thickness and the charge passed.

A pPy layer was electrodeposited on the Au surfaces by applying a constant oxidative potential. The chronoamperograms of pPy electropolymerization shown in Fig. 4(a) reveal the effect of surface conditions on the layer growth. When a bare Au or O_2_ plasma-treated Au electrode was used, the chronoamperometric curves showed a steep increase in the initial current, demonstrating that pPy deposited rapidly on these surfaces.

By contrast, when the PEI-O_2_ Au electrode was used, the chronoamperometric curves exhibited a gradual increase in the initial current, demonstrating that the growth of pPy is hindered by the electrically insulating PEI layer on the surface. Given that the typical electrodeposition process takes up to ~2000 s, the PEI layer on the Au surface would undergo local rearrangement of its repeat unit and become entangled with the extended chains of pPy via site/polymer diffusion^18^. The PEI layer impedes the initial growth of the pPy layer, so the deposition time required for fabricating a pPy layer on PEI-coated Au is longer than that required for a pPy layer on a bare electrode, given that the thicknesses are the same. However, the relationship between the chronometric charge and the thickness or the topology was unaffected by the presence of the PEI layer (Fig. 4(b)). The electrical conductivity of the pPy films was measured using the four-point probe method. Regardless of the presence of the PEI coating, the conductivity of the pPy films on Au electrodes was similar (~0.2 S/cm). This conductivity is within the range of values observed for pPy films (0.001 to 100 S/cm)^19,20^.

**Figure 5:**
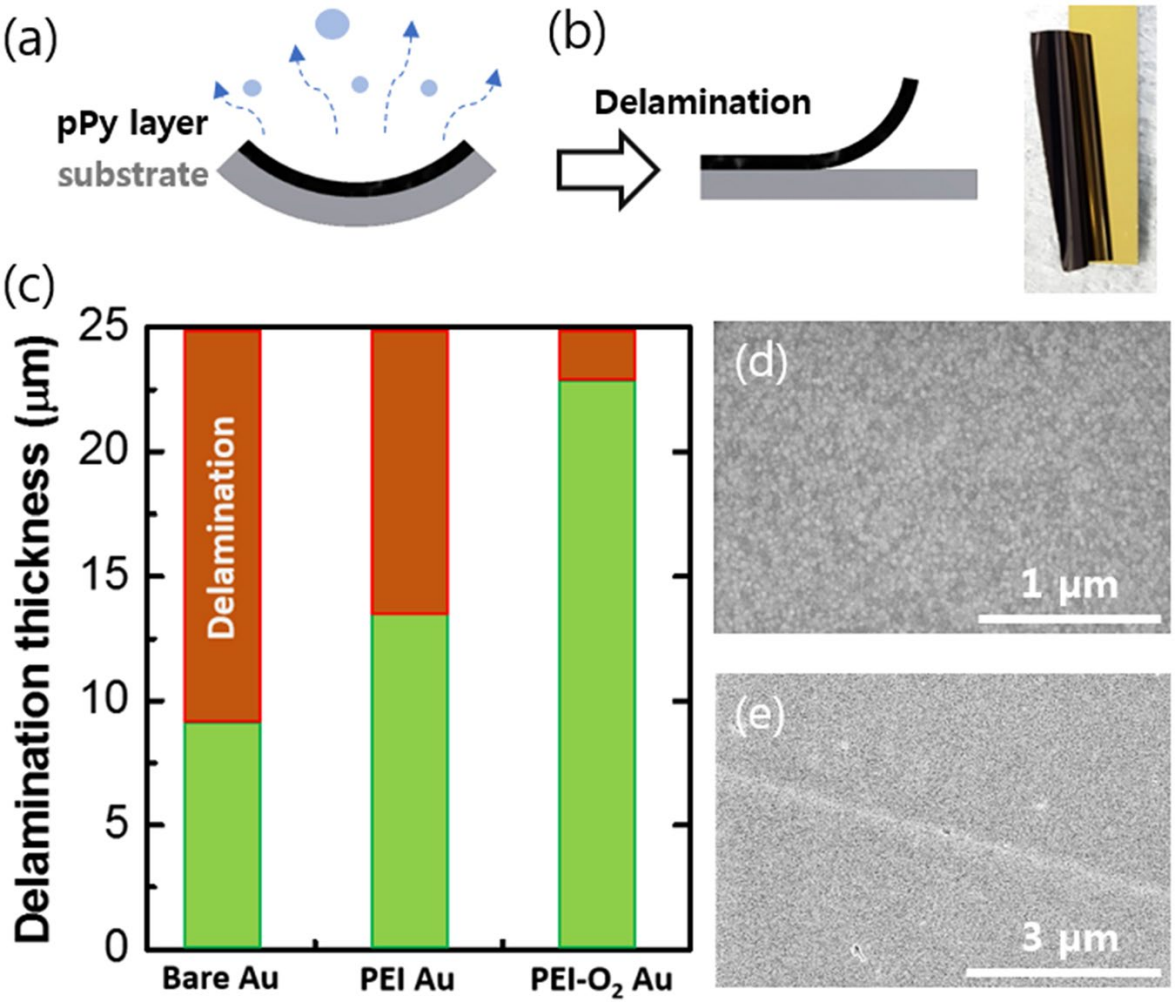
Schematic diagrams showing (**a**) the shrinkage of the pPy layer during drying and (**b**) the delaminated state of the pPy layer and its digital image (right). (**c**) Delamination thickness of pPy layers on various Au surfaces. SEM images of (**d**) Au surface and (**e**) pPy layer surface after its delamination.

Following pPy electropolymerization, the wet pPy layers were rinsed and dried in air. As water evaporates from the pPy layer, it shrinks and delaminates when its thickness is larger than a certain value (delamination thickness) for a given surface treatment and environment (e.g., humidity and temperature; Fig. 5(a,b)). Generally, the total strain energy released (Gc) during the delamination of a layer from a rigid substrate quantifies the interfacial adhesion and it is proportional to the delamination thickness^21^. Therefore, the delamination thickness represents a measure of the relative interfacial adhesion strength.

Figure 5(c) shows that the delamination thickness of the pPy layer on the bare Au electrode was 9 µm, but was ~23 µm (i.e., 250% higher) on the PEI-O_2_ Au electrode. Clearly, these results demonstrate that our proposed surface treatment method does indeed enhance the interfacial adhesion. Additionally, the bonding between the pPy layer and the PEI-O_2_ Au electrode was stronger than that with the PEI-coated Au without O_2_ treatment, confirming that polar groups on the O_2_-treated Au contribute to the enhanced interfacial adhesion. The positively charged pPy backbones may be attracted directly to the surface polar groups (e.g. C = O) of the O_2_-treated Au surface even though the surface is covered with the PEI layer. This is because the PEI layer is not defect-free, the high-molecular-weight branched PEI has a large free volume, and the mobility of the PEI segments during the electropolymerization might expose the bottom Au surface. This direct interaction would further enhance the adhesion. Furthermore, compared with the conditions of 30% relative humidity and 23 °C, the adhesion between the pPy layer and PEI-O_2_ Au remained twice as strong as that between the pPy layer and bare Au at 10% relative humidity and 60 °C (Fig. S2).

After delamination of the pPy layer from the PEI-O_2_ Au electrode, the Au surface and the delaminated side of the pPy surface were observed by SEM. Figure 5(d,e) reveals that no appreciable pPy or PEI residues were left on the Au surface after delamination; the electrode side of the pPy layer showed a smooth surface profile similar to that of the bare Au surface. These results confirm that the cohesion of the pPy layer is stronger than the interfacial adhesion between the pPy layer and PEI-O_2_ Au. The PEI patches seen in Fig. 3 disappeared following the pPy delamination, indicating that the interaction between PEI and the pPy layers is stronger than that between PEI and plasma-treated Au. The interatomic interactions between PEI and pPy might not be strong because both the electropolymerized pPy backbone and the adsorbed PEI are positively charged^11,22^. Therefore, the good adhesion between PEI and pPy layers might be due to other reasons, one of which would be the physical entanglement between the two entities. The pPy chains growing from the surface may be physically entangled with the high-molecular-weight branched PEI layer. The large free volume of PEI^23^ and some segments stretching out from the surface would contribute to this physical interaction.

**Figure 6:**
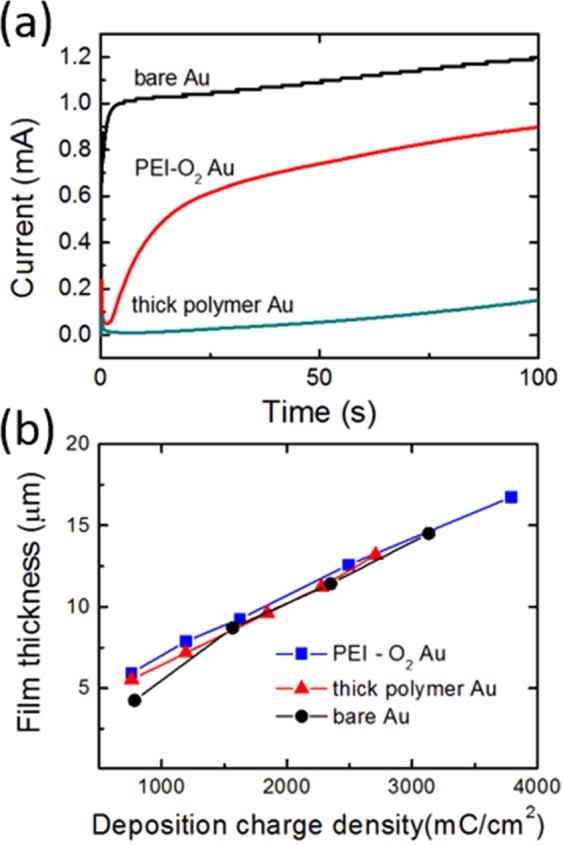
(**a**) Cyclic voltammograms of 4-μm-thick pPy layers on surface-treated electrodes: Scan rate = 1 mV/s, electrolyte = 0.2 M HCl. (**b**) Charge capacity retention of the pPy layers as a function of scan rate (mV/s).

Cyclic voltammetry was performed to determine the effects of the PEI layer on the electrochemical performance of the overlaying pPy layers. Figure 6(a) shows that all the cyclic voltammograms (CVs) of the pPy layers exhibit two faradaic peaks, which are indicative of a redox reaction of IC and are consistent with previous reports^11^. The CV corresponding to the pPy layer on a PEI-O_2_ Au electrode was almost identical to that of the pPy layer on the bare electrode, confirming that a thin PEI coating does not affect the electroactivities of the pPy layer and its dopant. Electrochemical impedance microscopy was used to investigate the electrochemical properties at different time scales (or frequency). Figure S3 shows the Bode plot of the pPy films on different Au electrodes. In the frequency range 1 to 10^5^ Hz, the impedance of the pPy film on PEI-O_2_ Au was similar to that on a bare Au, indicating that the electrochemical properties of the pPy film were not affected by the underlying PEI layer. This trend is in good agreement with the voltammograms shown in Fig. 6. The impedance data also indicate that the electrical and ionic conductivities of the pPy films on bare Au and PEI-O_2_ Au are identical, which is in agreement with the conductivity data mentioned earlier. This result is consistent with previous work, where a physisorbed layer of polypeptides had negligible effect on the electrode impedance^24^. A broader peak-to-peak separation and lower charge storage capacity were observed only for the sample comprising the pPy layers deposited on a thick LPEI/PAA coating.

Charge storage capacity per unit area (areal capacity, mC/cm^2^) of the pPy layer increased with increasing thickness, regardless of the surface pretreatments (i.e., PEI coating, O_2_ treatment). Therefore, in terms of achieving high capacity, using PEI-O_2_ Au electrodes is advantageous over using bare Au electrodes since thicker pPy layers can be fabricated on PEI-O_2_ Au electrodes. The maximum areal capacity of the pPy layer on the PEI-O_2_ Au electrode was more than twice as large as that of the pPy layer on the bare Au electrode: 538 mC/cm^2^ for 21-μm thick pPy layers on the PEI-O_2_ Au vs. 220 mC/cm^2^ for 8-μm thick pPy layers on the bare Au. This maximum capacity value of the pPy layer (i.e., 538 mC/cm^2^, 21 μm) is comparable with the high areal capacity electrodes with similar thicknesses: carbon nanotubes (~300 mC/cm^2^, 18 μm) on a porous carbon paper, pPy nanotubes (625 mC/cm^2^, 30 μm), and reduced graphene oxide/pPy films (~300 mC/cm^2^, 18 μm)^25–27^. In terms of the areal energy and power densities, the 21-μm thick pPy layer delivered 35 μWh/cm^2^ at 2.8 mW/cm^2^, which is comparable to the densities delivered by 18-μm reduced graphene oxide/pPy films: 60 μhWh/cm^2^ at 1 mW/cm^2^ ^28^. Figure 6(b) shows that, regardless of the presence of the PEI layer, the charge storage capacity of the pPy layers decreased by only 25% as the scan rate was increased from 10 mV/s to 1000 mV/s, showing that the PEI layer does not affect the retention capability of the pPy layer. These results demonstrate that using a PEI layer is effective for enhancing not only the adhesion but also the thickness-dependent performance of pPy electrodes.

In general, pPy doped with anions such as IC swells with negative potential sweep due to the influx of cations/water into the pPy matrix, and the degree of expansion increases as the potential becomes more negative^29^. The electrochemical cycle stability of pPy films is likely to be dependent on the applied potential window. As shown in Fig. S5, 7-µm-thick pPy films on both bare and PEI-O_2_ Au retained most of the initial capacity (>94%) after 500 cycles when subjected a moderate potential sweep from -0.2 to 0.3 V (vs. Ag/AgCl), demonstrating excellent electrochemical cycle stability. In this potential window, no delamination of the pPy films occurred on both surfaces.

**Figure 7:**
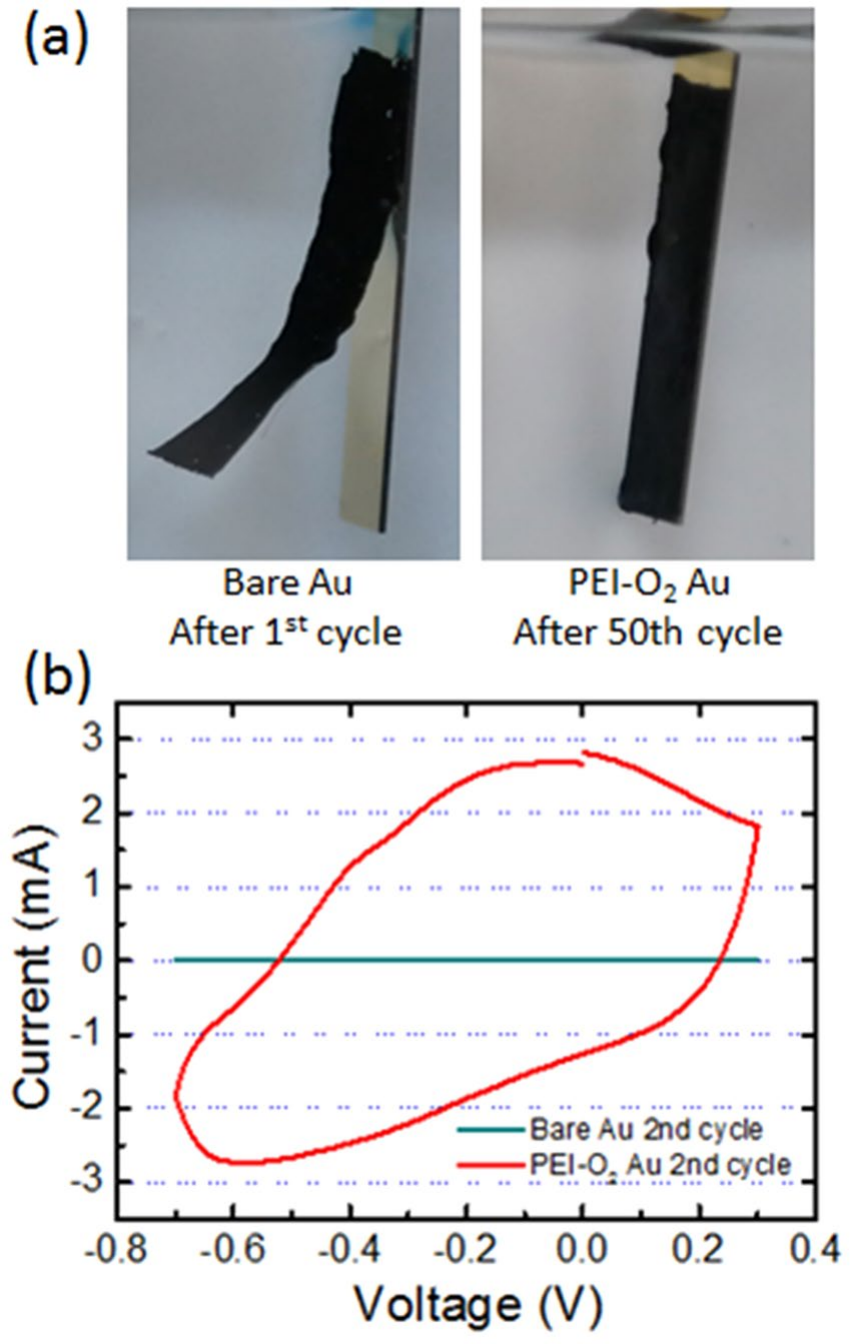
(**a**) Digital images of pPy films on bare PEI and PEI-O_2_ Au after 1^st^ cycle of a potential sweep from −0.7 to 0.3 V (vs. Ag/AgCl). (**b**) Cyclic voltammogram of pPy films on bare PEI and PEI-O_2_ Au. (scan rate = 20 mV/s, film thickness = 7 µm, in 0.1 M KCl solution).

However, under a more negative sweep from −0.7 to 0.3 V (vs. Ag/AgCl), the pPy films on the bare Au electrodes delaminated instantly at the first negative potential sweep, while those on the PEI-O_2_ Au electrode were well attached on the surface (Fig. 7(a)). Consequently, the charge stored in the pPy film on bare Au electrodes was not accessible for the second cycle, as indicated by the flat line in Fig. 7(b). In contrast, the electrochemical charge stored in the pPy film on the PEI-O_2_ Au electrode was not only accessible in the second cycle (red line in Fig. 7(b)) but also in the subsequent 100 cycles. These results demonstrate that our proposed surface treatment is beneficial for preventing the delamination during electrochemical processes, and therefore, is useful for realizing pPy films with good electrochemical cycle stability.

## Conclusion

In summary, this study introduces a simple method for enhancing the interfacial bonding between a pPy layer and the underlying electrode surface by introducing an adhesion-enhancing PEI layer. The PEI layer promoted the adhesion of the pPy layer by up to 250 % via interfacial interactions with the Au surface and by surface entanglement/interactions with the pPy layer. Importantly, this adhesion-promoting layer did not affect the physical and electrochemical performances of the overlaying pPy layer. Therefore, this strategy expands the range of the pPy layer thickness without delamination (e.g., from 9 to 23 μm at room temperature and relative humidity of 40 %), which is essential for fabricating high-performance electrodes for electrochemical and actuator applications. The areal capacity of the pPy layer was proportional to the thickness, and it reached up to ~530 mC/cm^2^. Moreover, the pPy layer did not delaminate from the surface when it was deposited on the PEI-coated Au electrode even at more negative potentials, where the pPy layers on the untreated surface tended to peel off from the surface due to electrochemical swelling. We believe that our strategy can be easily expanded to various combinations of CPs, dopants, and substrates for the development of stable, high-performance CP electrodes for energy devices and actuators.

## Supplementary information

Supporting Information
