# Publisher Correction: Increase in Interfacial Adhesion and Electrochemical Charge Storage Capacity of Polypyrrole on Au Electrodes Using Polyethyleneimine

**DOI:** 10.1038/s41598-019-42219-5

**Published:** 2019-04-16

**Authors:** Kyung-Geun Kim, Sung Yeol Kim

**Affiliations:** School of Mechanical Engineering, Kyungpook National University, Daegu, 702-701 Republic of Korea

Correction to: *Scientific Reports* 10.1038/s41598-019-38615-6, published online 18 February 2019

In this Article, Figure 6 is a duplication of Figure 4. The correct Figure 6 appears below as Figure 1.

**Figure 1:**
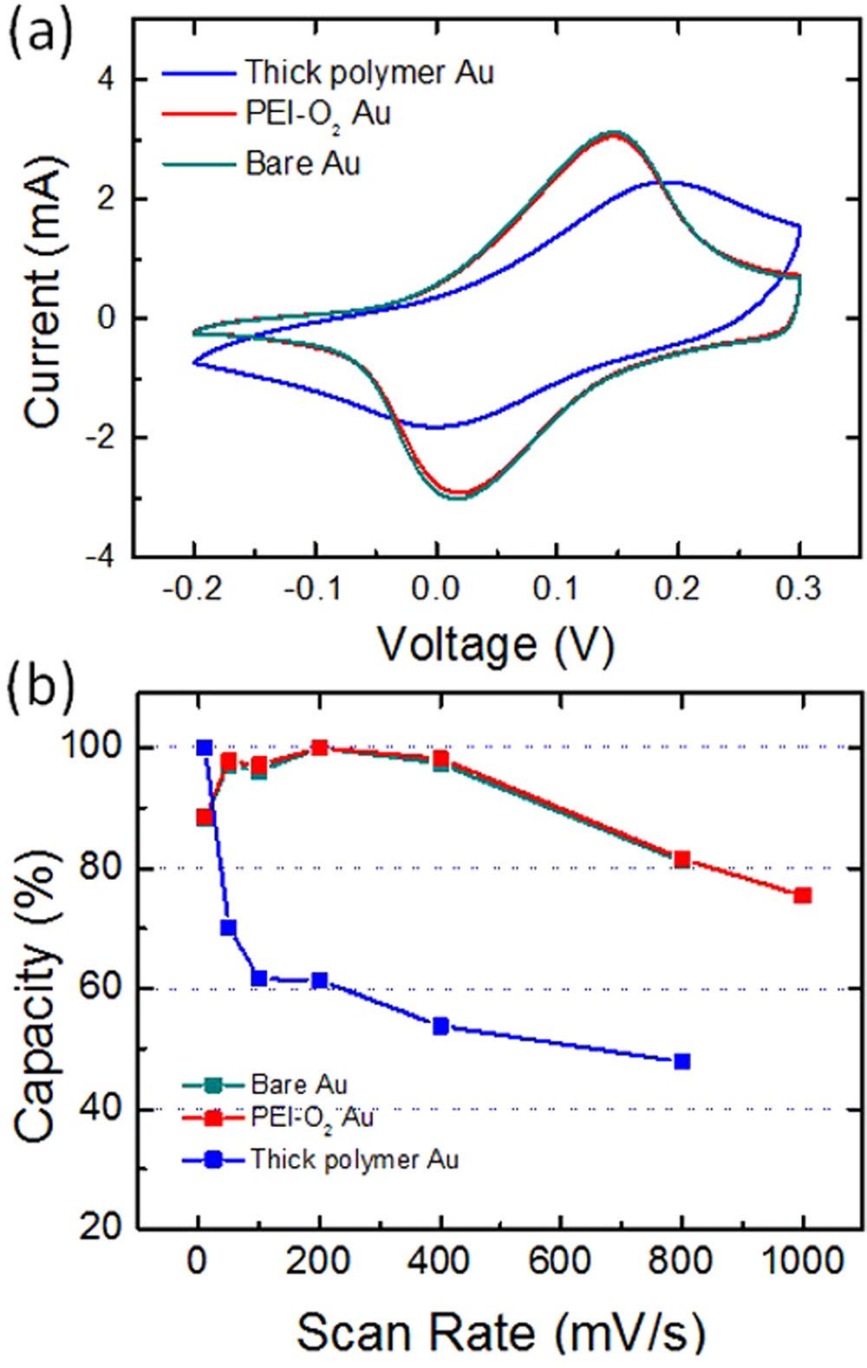
(**a**) Cyclic voltammograms of 4-μm-thick pPy layers on surface-treated electrodes: Scan rate = 1 mV/s, electrolyte = 0.2 M HCl. (**b**) Charge capacity retention of the pPy layers as a function of scan rate (mV/s).

