# Supplementary material for: Increase in Interfacial Adhesion and Electrochemical Charge Storage Capacity of Polypyrrole on Au Electrodes Using Polyethyleneimine
Source: Sci Rep. 2019 Feb 18;9:2169. doi: 10.1038/s41598-019-38615-6 (PMC6379486; doi:10.1038/s41598-019-38615-6)
Supplement: Supplementary file 1 — Supporting Information [file 41598_2019_38615_MOESM1_ESM.docx]

Supporting Information

Increase in Interfacial Adhesion and Electrochemical Charge Storage Capacity of Polypyrrole on Au Electrodes Using Polyethyleneimine

Kyung-Geun Kim and Sung Yeol Kim*

School of Mechanical Engineering, Kyungpook National University, Daegu 702-701, Republic of Korea

*Corresponding Author. E-mail: [sykim.knu@gmail.com](mailto:Tayhas_Palmore@brown.edu)


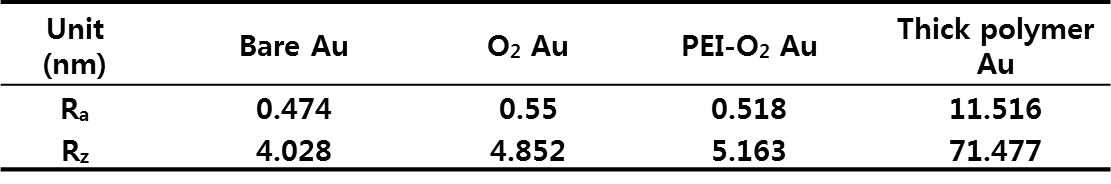


**Table S1.** Surface roughness as a function of surface treatments


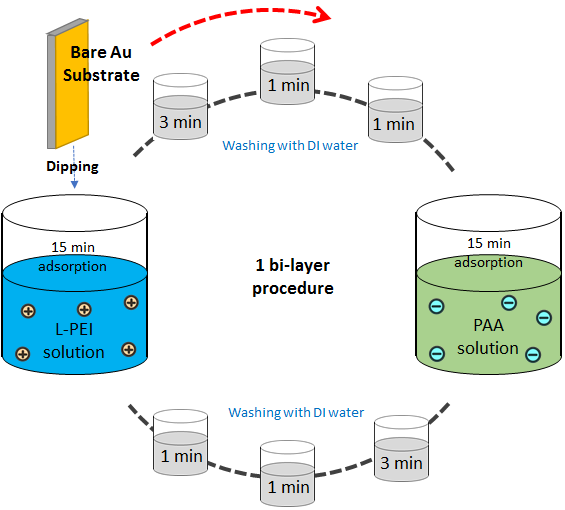


**Figure S1.** Schematic diagrams of the layer by layer assembly process


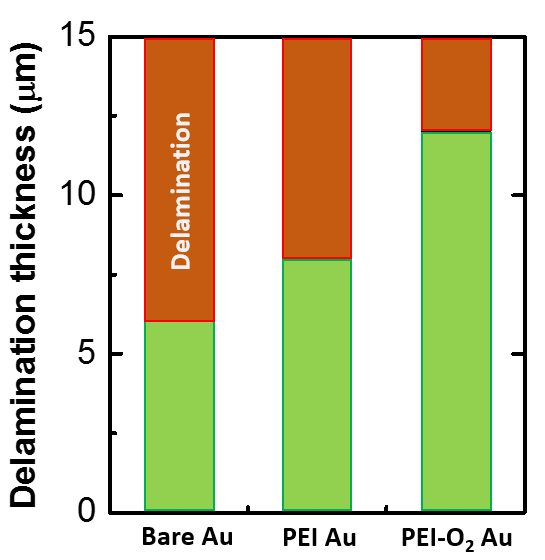


**Figure S2.** Delamination thickness of pPy layers on different Au electrodes at a temperature of 60 °C and a relative humidity of 10%.


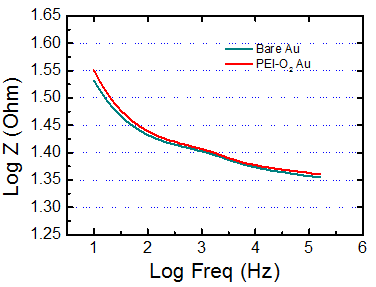


**Figure.S3** Impedance of pPy films (thickness =7 µm) in 0.1 M KCl solution. (scan from 1 MHz to 10 Hz, Applied voltage =0.16 V vs Ag/AgCl reference electrodes)


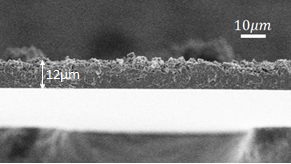


**Figure S4.** SEM image of the cross section of a pPy film.


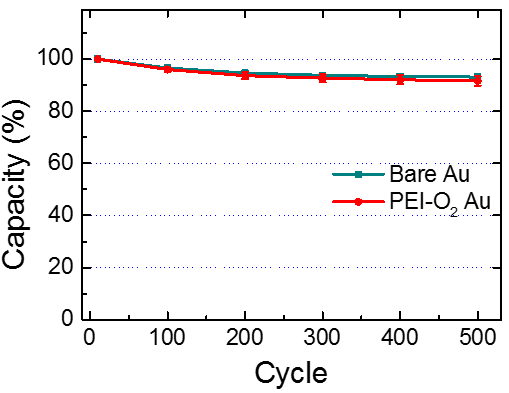


**Figure S5.** Charge storage capacity of pPy films as a function of electrochemical cycle from -0.2 to 0.3 V (vs. Ag/AgCl) (Scan rate = 100 mV/s, film thickness = 7 µm in 0.2 M HCl solution)
